# The Signature Amino Acid Residue Serine 31 of HIV-1C Tat Potentiates an Activated Phenotype in Endothelial Cells

**DOI:** 10.3389/fimmu.2020.529614

**Published:** 2020-09-25

**Authors:** Malini Menon, Roli Budhwar, Rohit Nandan Shukla, Kiran Bankar, Madavan Vasudevan, Udaykumar Ranga

**Affiliations:** ^1^Jawaharlal Nehru Center for Advanced Scientific Research, Bangalore, India; ^2^Bionivid Pvt. Ltd., Bangalore, India

**Keywords:** HIV-1C, genetic subtypes, Tat, signature amino acid residue, CCL2, endothelial cells, angiogenesis

## Abstract

The natural cysteine to serine variation at position 31 of Tat in HIV-1C disrupts the dicysteine motif attenuating the chemokine function of Tat. We ask if there exists a trade-off in terms of a gain of function for HIV-1C Tat due to this natural variation. We constructed two Tat-expression vectors encoding Tat proteins discordant for the serine 31 residue (CS-Tat vs. CC-Tat), expressed the proteins in Jurkat cells under doxycycline control, and performed the whole transcriptome analysis to compare the early events of Tat-induced host gene expression. Our analysis delineated a significant enrichment of pathways and gene ontologies associated with the angiogenic signaling events in CS-Tat stable cells. Subsequently, we validated and compared angiogenic signaling events induced by CS- vs. CC-Tat using human umbilical vein endothelial cells (HUVEC) and the human cerebral microvascular endothelial cell line (hCMEC/D3). CS-Tat significantly enhanced the production of CCL2 from HUVEC and induced an activated phenotype in endothelial cells conferring on them enhanced migration, invasion, and *in vitro* morphogenesis potential. The ability of CS-Tat to induce the activated phenotype in endothelial cells could be of significance, especially in the context of HIV-associated cardiovascular and neuronal disorders. The findings from the present study are likely to help appreciate the functional significance of the SAR (signature amino acid residues) influencing the unique biological properties.

## Introduction

HIV-1 genetic subtypes differ from one another in several molecular and biological properties, including genetic sequence variation, co-receptor usage, pathogenicity, replication fitness, transmission, and geographical distribution (1, 2). While factors such as the demographic variations, founder effect, host factor landscape differences, and socioeconomic parameters could significantly influence and modulate the observed differences among the HIV-1 genetic subtypes, natural genetic variations unique for individual viral subtypes play critical roles (3). The examination of the inherent genetic traits of viral subtypes could offer important clues to the survival advantage that the viral strains may have acquired in a specific population. Among several genetic subtypes of human immunodeficiency virus type I (HIV-1), subtype C (HIV-1C) is responsible for nearly half of HIV-1 infections worldwide (4). Signature sites are specific positions in an amino acid or nucleic acid alignment of variable sequences that are distinctly representative of a query set (viral genetic subtype), relative to a background set (constituted by the other genetic subtypes) (5). In other words, signature amino acid residues (SAR) are unique to one or a small number of viral genetic subtypes, present in a majority of the viral strains of the genetic subtypes, and either absent or present in only a small minority of other genetic subtypes. The study of the signature amino acid residues may offer valuable leads underpinning the biological differences among genetic subtypes of HIV-1 or other viruses and the survival advantage that the viral strains may have acquired in a specific population. In the present study, we used HIV-1 Tat as a model protein to understand the significance of S_31_, an important SAR of HIV-1C.

HIV-1 Tat regulates several functions critical for viral survival (6, 7) and modulates several host signaling pathways (8–10). Thus, the pleiotropic nature of Tat biology offers a variety of endpoints to examine the impact of SAR on the biological functions of Tat. The previous work from our laboratory identified seven SAR in HIV-1C Tat comprising H_29_, S_31_, L_35_, Q_39_, S_57_, P_60_, and E_63_ (11). Of these SARs, the biological significance of one of the SARs, S_31_, was experimentally evaluated in great detail (11–13). The substitution of a cysteine residue with a serine in HIV-1C, disrupts the dicysteine motif (C_30_C_31_), consequently attenuating the chemokine function of Tat (11–13). Our previous work, however, did not explore the evolutionary advantage of the natural variation of Tat, cysteine to serine substitution, unique to HIV-1C. In this backdrop, we investigated the functional significance of SAR S_31_ in HIV-1C Tat in the present work.

Several previous reports, including from our laboratory, suggested a neuroprotective function for the natural polymorphism of C31>S in HIV-1C Tat (11, 13–15). Li et al. examined if the interaction of Tat with the NMDA receptor, a key receptor involved in neurological processes such as learning, memory, and excitotoxicity, displayed subtype-specific differences. They found a significantly reduced neurotoxicity of C-Tat as compared to B-Tat, with the primary factor underlying this difference being the presence of C31 in B-Tat as against S31 in C-Tat (14). The residue, C31, forms a disulfide bond with the NR1 subunit of the NMDA receptor leading to activation. The presence of S31 in C-Tat attenuates this interaction and results in reduced neurotoxicity. These findings were corroborated by Mishra et al. when they demonstrated significantly reduced levels of apoptosis, chemokine secretion, and oxidative stress in the primary neurons and astrocytes exposed to C-Tat as compared to B-Tat (13). Further, Rao et al. demonstrated that the Tat dicysteine motif (C30C31) of C-Tat to function as the primary determinant of neurovirulence using a SCID mouse HIV-encephalitis model. The conclusions were drawn based on the monocyte migration, neurovirulence, and cognitive defects manifested following intracranial injection of MDM infected with Southern African HIV-1C isolate (from Zambia; 1084i) or HIV-1C isolate (Indie C1 from Southeast Asia) or an HIV-1B isolate (ADA from the US) (15). The most extensively examined impact of Tat-mediated EC activation and dysfunction in HIV-1 infection is in the context of HIV-associated neurocognitive disorders (HAND) and HIV-associated cardiovascular or angio-proliferative disorders (including Kaposi's sarcoma). In the context of the Tat dicysteine motif, reports from the South African population are of relevance (16). In South Africa, although the most prevalent viral strain is HIV-1C, a significant proportion of Tat in these viruses contains the intact C30C31 dicysteine motif. The results from the study suggested that the C31S substitution does not confer decreased impairment in cognitive performance. This study contradicted the earlier studies reporting substantial neurocognitive impairment in an HIV-1C-infected population despite the presence of the disrupted C30C31 motif in the Tat protein (17, 18).

Several studies previously attempted to compare the impact of the Tat proteins of HIV-1B and HIV-1C origin in modulating the functions of the host cell (1, 13, 19, 20). The HIV-1B Tat is seen to exhibit the superior potential to cause neuronal toxicity and synaptic plasticity, induce apoptosis in human primary neurons, and augment a pro-inflammatory cytokine response. The present study differs in important technical aspects from the previous reports by focusing on detecting the early intracellular events of Tat induction in certain respects, importantly, in exercising a tight control on Tat expression. Tat can modulate cell functions as an intracellular viral factor or when secreted into extracellular fluids as an extracellular protein (21–24). Tat, a powerful immunomodulator, can alter the expression of several cellular genes, including that of potent pro-inflammatory cytokines (21, 25, 26). The secondary amplification of cellular signaling events by pro-inflammatory cytokines and other immunomodulatory molecules could lead to a cascading effect, often masking of the primary impact of Tat. To detect only the early cellular events of Tat induction, and avoid the interference due to the secondary amplification of cellular signaling events, an efficient control on Tat expression was necessary.

To this end, we engineered Jurkat cells to express two variant forms of Tat—containing a serine (CS-Tat, wild type of HIV-1C) or a cysteine (CC-Tat, common in all the non-HIV-1C strains) residue at position 31, otherwise identical at all other positions. To detect only the early cellular events of Tat induction, we placed Tat expression under an inducible Tet-On promoter and generated stable Jurkat T-cells. Following induced expression of variant Tat in Jurkat cells, we evaluated the early cellular gene expression using RNA-Seq. The RNA-Seq analysis was suggestive of significant enrichment of the pathways and gene ontologies pertaining to angiogenic signaling events by CS-Tat, and the subsequent validation of these signaling events was executed using human umbilical vein endothelial cells (HUVEC) and the human cerebral microvascular endothelial cell line (hCMEC/D3).

## Methods

### Cell Culture

The Jurkat cells (Clone E6-1) were procured from ATCC and cultured in RPMI 1640 (AL162S, HiMedia Laboratories, Mumbai, India), supplemented with 10% fetal bovine serum (10082147, Thermo Fisher Scientific, Waltham, USA), 2 mM L-glutamine (G8540, Sigma, St. Louis, USA), 100 units/ml penicillin G (P3032, Sigma, St. Louis, USA), and 100 μg/ml streptomycin (S9137, Sigma, St. Louis, USA). The HUVEC cells procured from ATCC, Virginia, USA, were used for the biological assays at passages 4 to 6. The cells were cultured in Ham's F-12K (Kaighn's) Medium (21127-022, Thermo Fischer Scientific), supplemented with 10% fetal bovine serum, 0.1 mg/ml heparin (H3149-100 KU, Sigma), and 0.03 mg/ml endothelial cell growth supplement, ECGS (E2759, Sigma). The HUVEC were seeded in tissue culture dishes coated with 0.1% gelatin (TCL059, HiMedia Laboratories, Mumbai, India). The immortalized HCMEC/D3 cells, procured from Cedarlane Cellutions Biosystems Inc. (CLU512), were cultured using EndoGRO MV complete media kit (SCME004, Millipore), on culture dishes coated with 150 μg/ml of type-I collagen from Rattail (C3867, Sigma). The Human Embryonic Kidney 293T (HEK293T) cells were procured from ATCC and cultured in Dulbecco's Modified Eagle's Medium (D1152, Sigma, St. Louis, USA), supplemented with 10% FBS, 2 mM L-glutamine, 100 units/ml penicillin G, and 100 μg/ml streptomycin.

### Construction of Expression Vectors

To establish the doxycycline-inducible expression system, the Tet-inducible Tat-expression cassettes were cloned into the third-generation HIV-1-based lentivector expression vector, pCDH-CMV-MCS-EF1-copGFP (CD511B-1, System Biosciences, California, USA). The reverse tetracycline-controlled transactivator 3 gene (rtTA3) was amplified from pTRIPZ (GE Life Sciences, Chicago, USA) (FP: AGTTATGAATTCATGTCTAGGCTGGACAAGAGC and RP: ATACGAGGATCCTTACCCGGGGAGCATGTCAAGGTC). The second-generation Tet-On promoter or tetracycline response element (TRE), Ptight, was amplified from the commercial vector pTRE-Tight (631059, Clontech, California, USA) (FP: TAGGCGATTAATGAGGCCCTTTCGTCTTCACTC, RP: CCATGGGCTAGCCCGGTCCAGGCGATCTGACGG). The BL43 WT C-Tat (CS) and its SAR variant (CC) Tat were amplified from vectors p214.CS and p214.CC, respectively, generated previously in the laboratory and cloned into the MCS downstream of the TRE promoter using the enzyme sites, BglII and EcoRI for CS-Tat and SalI and BamHI for CC-Tat. The recombinant clones express the WT (CS) and variant (CC) Tat proteins, under the inducible Tet-responsive promoter, TRE.

### Generation of Jurkat-Tat Stable Cell Lines for Inducible Tat Expression

To exercise tight control on the intracellular expression, we engineered the Tat expression under the control of a second-generation tetracycline/doxycycline-inducible promoter (Tet-On promoter). We established the Tet-On system for Tat expression by cloning two independent expression cassettes into the lentiviral expression vectors. The first lentiviral expression vector was designed to constitutively express rtTA3 and the puromycin resistance gene from a cytomegalovirus (CMV) promoter. The second vector was engineered for the inducible expression of the variant Tat proteins (Tet-On CS- or CC-Tat) from the Tet-On promoter. Additionally, an empty vector (Tet-On EV) that does not express Tat was also generated to serve as a control in all experiments. Of note, these vectors constitutively expressed the copepod green fluorescent protein (copGFP) as a reporter from the EF1α promoter. The transduced Jurkat cells were allowed to stabilize for 10 days following infection. The cells were subsequently analyzed using a flow cytometer for copGFP expression. The CS, CC, and EV Jurkat stable cells were established using fluorescence-assisted cell sorting (Supplementary Figure 1A).

The lentiviral vectors were used in combination with the packaging vectors (pVSV-G, pCMV-Rev, and psPAX2) to generate VSV-G pseudotyped lentiviruses in HEK293T for transduction. The Tat-stable cell lines were generated in two successive rounds of transduction. In the first round, the Jurkat cells were transduced with pseudotyped CMV-rtTA3-IRES-puromycin viruses (100 ng/ml p24 equivalent) for stable expression of rtTA3 and selected using puromycin. The rtTA3 expression from the transduced cells was confirmed using a quantitative PCR (FP: ACCCGCCCAACAGAGAAACAGTACGAAACC, and RP: CCTGTTCCTCCAATACGCAGCCCAGTGTAAAG) and stable cells were selected at a drug concentration of 800 ng/ml of puromycin. Several rtTA3 clonal cell lines were established by limit dilution and screened for the dox responsiveness in a separate experiment. The clone demonstrating the highest level of dox induction (~180-fold induction, following 12 h of induction) was selected for the subsequent Tat stable transduction. In the second round, the stable rtTA3-Jurkat cell line was transduced with the pseudotyped parental virus TRE-EF1α-copGFP (EV) or the Tat-expression variants (CS-Tat or CC-Tat). The rtTA3-Jurkat cells were infected with Tat viruses generated in HEK293T cells (the equivalent of 1.0, 0.1, and 0.01 ng/ml of p24 varying p24), and the expression of GFP was monitored using a flow cytometer. The viral infection at 0.1 ng/ml generated approximately 5% of GFP-positive cells, representing a single integration event per cell statistically (27). Using these conditions of viral infection, we established Tat-stable cell populations by GFP sorting. Optimal expression of Tat was induced from stable cell lines at a doxycycline concentration of 800 ng/ml. The expression of Tat transcript was quantitated employing a quantitative PCR (FP: GGAATCATCCAGGAAGTCAGCCCGAAAC, and RP: CTTCGTCGCTGTCTCCGCTTCTTCCTG).

### The Real-Time PCR Analysis for Viral Integration

Real-time PCR of the LTR was used to confirm a comparable number of integration events in the Tet-On Tat stable cells. Ten nanogram of genomic DNA isolated from the cells were amplified in a real-time PCR using primers targeting the strong-stop negative-strand DNA in the viral LTR. A serial 10-fold dilution of LTR copies from the genomic DNA extracted from TZM-bl cells (that contains two copies of stably integrated reporter provirus) was used in the LTR real-time PCR (FP: GATCTGAGCC(T/C)GGGAGCTCTCTG, and RP: TCTGAGGGATCTCTAGTTACCAGAGTC). A standard curve was constructed using serially diluted plasmid DNA containing HIV-1 LTR and to assess the sensitivity of the real-time PCR. C_t_ values of 24.07, 24.10, and 24.13 cycles were obtained for the CS, CC, and EV cell populations, respectively (Supplementary Figure 1B) suggesting comparable frequency of integration (28). We used glyceraldehyde-3-phosphate dehydrogenase (GAPDH) reference gene PCR for normalization of the real-time PCR data (22.79, 22.56, and 22.99 C_t_ values, respectively).

### The NGS Workflow

We used the Illumina HiSeq 2000 platform to perform the RNA-Seq analysis and the TruSeq RNA Library preparation v2 kit (Illumina, California, USA) for library preparation. The reads of 100-bp length were obtained using a paired-end sequencing protocol. The tools used for resequencing analysis included- NGS QC Toolkit, TopHat, Cufflinks, DESeq, Microsoft Excel, and Cluster 3.0. The NGS QC Toolkit v2.3 was used for the quality control of the raw read data to obtain high-quality filtered reads. The high-quality filtered reads were aligned to the hg19 reference genome with TopHat (version 1.3.1). The gene model annotations and the known transcripts were deciphered using Hg19 assembly platform (UCSC genome browser). After the alignment to the reference genome, each RNAseq library was subjected to expression profiling using the tool package Cufflinks (version 1.1.0) (24). Differential gene expression profiling and differential analyses of the samples were performed for CS-Tat, CC-Tat, and EV (No Tat) using DESeq. Differentially expressed genes (DEGs) between the groups were calculated using the DESeq package in R version 3.2.5 (29). The threshold for DEGs was set as |log_2_ fold change| ≥1.0 and ≤ −1.0 and *p* ≤ 0.05. We computed both *p*-value and p-adjusted value (q-value) as a part of the standard analysis. To estimate the degree of biological variations between the replicates and to identify differentially expressed transcripts with acceptable false discovery rate, we proceeded with *p*-value as FDR to measure the total number of biologically meaningful differentially expressed genes (30). Identification of unique and overlapping genes within the DEG datasets was determined using Venny (bioinfogp.cnb.csic.es/tools/venny/index2.0.2.html). The differential gene lists were then subjected to perform significant biology wherein GO-Elite v1.2 (25) was used for Enrichment.

### Neutralization of Tat From the Jurkat-Tat Conditioned Medium

The conditioned media were harvested from the dox-induced Jurkat cells by centrifuging the cells at 1,500 × g for 5 min. One ml of conditioned media was incubated with one μg of E2.1 anti-Tat mouse monoclonal antibody (raised in-house) or IgG1 isotype control antibody (5415, Cell Signaling Technologies, Massachusetts, USA) for 30 min at room temperature with gentle agitation. The treated conditioned media were used in the assays.

### The Tube Formation Assay

The endothelial cell tubulogenesis was assayed using the growth factor reduced Matrigel-basement membrane matrix (354230, BD Biosciences). The Matrigel was spread evenly in wells of a 96-well plate; after 30 min of incubation at 37°C, HUVEC were suspended in the conditioned medium, seeded (0.12 million cells per ml) in the wells, and incubated for 24 h. The tube formation by HUVEC on Matrigel was assessed under an Olympus Inverted Microscope (100× magnification) and imaged at an interval of every 2 h. For the quantification of tube formation, the number of branch points of the formed tubes and other characteristics was evaluated using the Angiogenesis Analyzer plugin of the ImageJ software (31). Using this software, the original images (as captured at 100× magnification, three images per group) were transformed into the binary tree images and were treated with the “fill hole” method. The fill hole method minimizes the shadow effects in the image for quantification. Then, the images were skeletonized, and the binary trees were analyzed, and all the values were normalized with the analyzed area, making the area pixel number 19,20,000. Among several categories of quantification, the dimensional parameters such as a change in the size of the capillary-like network characterized by the number of nodes, number of junctions, number of segments, and number of branches were chosen for analysis. Since dimensional parameters do not fully characterize the architecture of the tubule patterns, the topological parameters, including total tubule length, total branching length, and total segment length, were also analyzed and compared among different groups (32, 33).

### The Transwell Migration Assay

The polycarbonate membranes (8 μm pore size) in the transwells of a 24-well tissue culture plate (CLS3413, Sigma) were pre-coated with Matrigel (growth factor reduced basement membrane matrix; diluted to 300 μg/ml in a serum-free medium; 100 μl per transwell). The plates were incubated for 2 h at 37°C, and HUVEC were seeded at a density of 0.5 million cells per ml in the transwells (100 μl per transwell) in complete media. The Tat-conditioned media were added to the lower compartment of appropriate transwells. After 3–12 h of incubation at 37°C, the upper surface of the filter was scraped away with a cotton swab, the filters were fixed and stained with Giemsa stain (38723, Fischer Scientific), and five fields per well in duplicates were imaged at 200 × magnification (34). The images were quantified for the number of migrated cells using ImageJ image processing software suite from NIH. The transwell counter plugin of ImageJ was used for quantification, which allows for high-throughput automated counting of the number of cells on membranes in Transwell invasion and migration assays (35).

### The Trans-Endothelial Permeability Assay

The trans-endothelial permeability of the hCMEC/D3 monolayer was assessed using 40 kDa FITC-Dextran (FD40, Sigma). The hCMEC/D3 cells were seeded on type-I collagen-coated 0.4-μm culture inserts and allowed to grow to confluence over 5 to 7 days. The confluent monolayers of cells were serum-starved for 12 h before the assay. The Jurkat-Tat conditioned media (12 h Dox-induced) were added to the confluent hCMEC/D3 monolayer on the inserts and wells. Following 24 h of incubation at 37°C, 0.1 μg/ml of FITC-dextran (40 kDa) was added apically to the inserts in the complete culture medium. The endothelial permeability was assessed by monitoring the flux of FITC-dextran across the endothelial monolayer by measuring the FITC-dextran fluorescence intensity in the spent media in the wells at regular intervals using a Fluorescence intensity spectral scanner (Varioskan Flash, Thermo Scientific).

### The Monocyte-Endothelial Cell Adhesion Assay

The hCMEC/D3 endothelial cells were grown to confluence on collagen-coated coverslips in 24-well plates. Following 12 h of serum starvation, the monolayer was exposed for 24 h to Jurkat-Tat conditioned media (12 h Dox-induced). In parallel, the THP1 cells were treated with the Jurkat-Tat conditioned media for 12 h, washed with HBSS (14175-079, Thermo Fischer Scientific), and labeled with 5 μM CalceinAM (L3224, Thermo Fischer Scientific), for 30 min. The CalceinAM-labeled THP1 cell suspension was added at 0.5 million cells per treatment to the treated endothelial monolayer. Following 10 h of the addition of the labeled THP1 cells, the coverslips containing the endothelial monolayer were washed with HBSS. The adhered THP1 cells under each treatment were imaged using a fluorescence microscope.

### Statistical Analysis

All the statistical analyses were carried out using GraphPad Prism Version 5.0 for Windows, GraphPad Software, La Jolla, California, USA. The data sets were analyzed using one-way or two-way ANOVA with relevant post-tests for multiple comparisons. All the multiple comparison tests were based on the assumption that the values after each treatment were randomly drawn from populations with the same amount of scattering. One-way ANOVA was used where three or more groups were compared followed by the use Tukey–Kramer test for multiple comparison tests to compare all pairs of columns and find which groups are different from which other groups. The Tukey–Kramer test in GraphPad includes the extension by Kramer to allow for unequal sample size. Two-way ANOVA was used when a response was affected by two or more factors. GraphPad Prism suggests the use of the Bonferroni method of post-tests following two-way ANOVA. The Bonferroni correction lowers the *P*-value that is considered to be significant to 0.05 divided by the number of comparisons. This correction ensures that the 5% probability applies to the entire family of comparisons, and not separately to each comparison. For the differential gene expression analysis in NGS, both *p*-value and p-adjusted values (q-value) were computed as a part of the standard analysis. To estimate the degree of biological variation between replicates and to identify differentially expressed transcripts with acceptable false discovery rate (FDR), the *p*-value as FDR was used to measure the total number of biologically meaningful and differentially expressed genes (30). Transcripts with |log_2_ fold-change| ≥1.0 and ≤ −1.0 and *p* ≤ 0.05 were considered statistically significant.

## Results

### RNA-Seq Analysis Following Tat Induction in Jurkat-Tat Stable Cells

**Figure 1:**
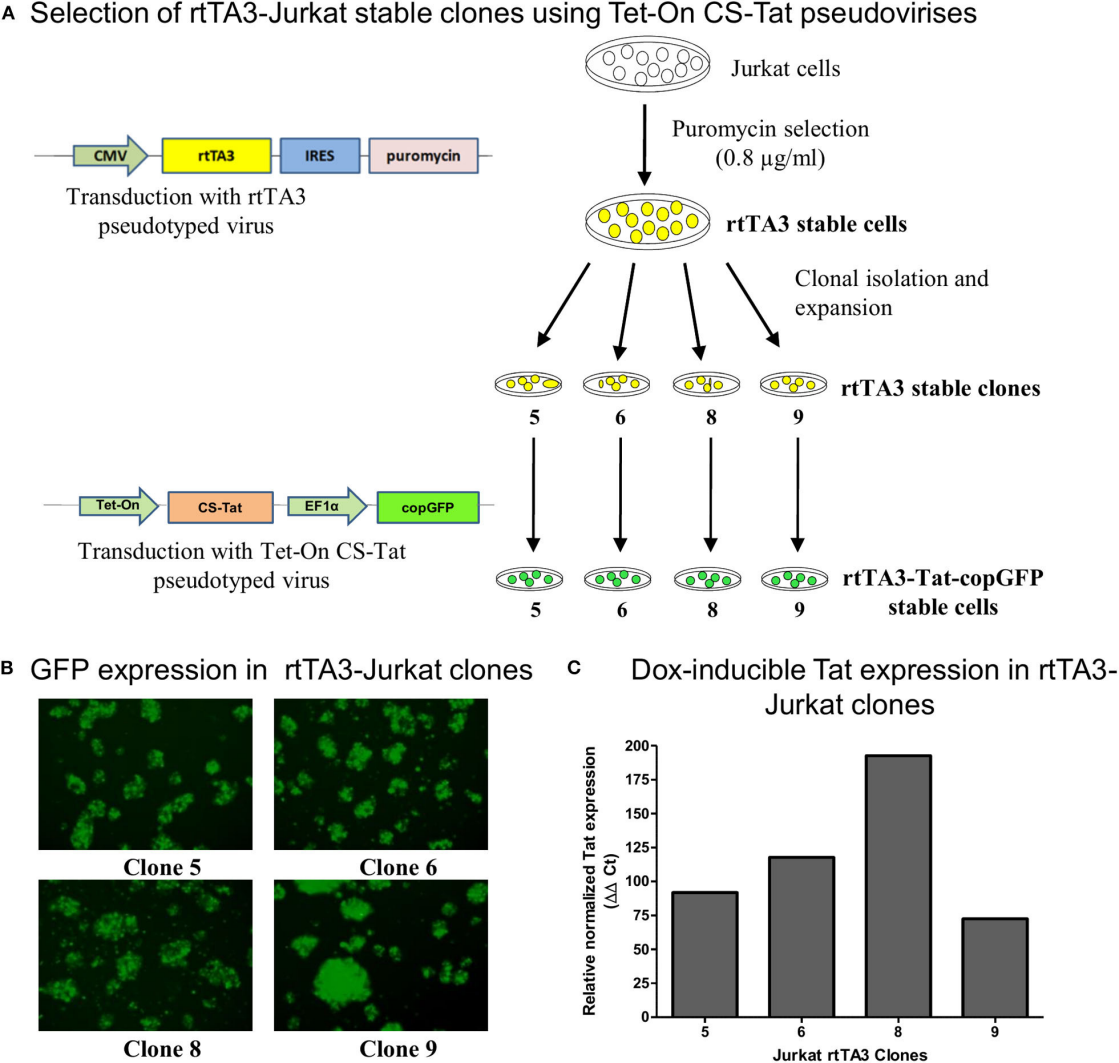
Generation and screening of rtTA3 stable cell lines. The Tat stable cells were obtained in two successive steps. **(A)** First, the Jurkat cells were transduced with the CMV-rtTA3-IRES-puromycin-pseudotyped lentivirus, and a pool of stable cells was obtained by selection with puromycin. The rtTA3-puro stable cells were subsequently subjected to limit dilution to obtain clonal cell lines, and each clone was infected with 30 ng/ml (p24 equivalent) of Tet-On CS-Tat EF1α-copGFP-pseudotyped virus. **(B)** The cells were imaged for copGFP expression at 72 h post-infection. **(C)** Each clonal population was subsequently treated with 800 ng/ml of Dox. A “No-Dox” control for each was used to evaluate the fold increase in Tat gene expression of each clone following doxycycline treatment. The cells were harvested at 24 h following Dox treatment, cDNA was synthesized from total cellular RNA, and a real-time PCR assay was performed to quantitate Tat transcripts. GAPDH was used as a reference gene control for normalization. The relative normalized Tat expression (using ΔΔCt method) was plotted.

Using lentiviral vectors, we established stable Jurkat cells expressing CS-Tat or CC-Tat under the control of the Dox-inducible system (Figure 1). To examine the primary events of Tat-induced host gene modulation, it was necessary to understand how soon the Tat transcripts are generated following Dox-induction. In a pilot analysis, we found 12 h of induction with 800 ng/ml of doxycycline to be optimal to detect Tat-induced early events (Supplementary Figure 2A). To examine the primary events of Tat-induced host gene modulation, it was necessary to understand how soon Tat transcripts are generated following Dox treatment of the cells. RNA extraction at the earliest time point of Tat induction is expected to be ideal for the whole transcriptome analysis (RNA-Seq). To this end, we induced rtTA3-CC-Tat Jurkat cells using 800 ng/ml of doxycycline (optimized using a Dox-dose response curve) and RNA was isolated from the cells at 6-h time intervals to monitor the modulation in the expression of Tat and a few cytokines comprising of TNF-α, IL-8, and IL-10 (Supplementary Figure 2A). Given that the gene expression of two important Tat-responsive genes, TNF-α and IL-8, peaked at 6–12 h, we selected the 12 h time point for the subsequent analyses. We confirmed similar levels of Tat expression at the RNA level in both CS and CC-Tat Jurkat cells using quantitative real-time PCR (*p* = 0.525, ns; Supplementary Figure 2B). Additionally, the modulation in the transcript level of cytokine/chemokine genes in CS and CC-Tat Jurkat cells was evaluated using gene-specific primers in a real-time PCR at a 12-h time point following Dox induction (Supplementary Figure 2C). These cytokines/chemokines are among many known to be modulated directly by Tat expression (20, 23, 36). Using the optimized experimental conditions, we performed the RNA-Seq analysis to gain a comprehensive overview of the global gene modulation following the induction of Tat variants in stable Jurkat cells (Supplementary Figure 3, and Supplementary Table 1). The RNA-Seq data of all the samples have been submitted to NCBI ((NCBI SRA accession number SUB7165990); http://www.ncbi.nlm.nih.gov/geo/query/acc.cgi?acc=GSE89266). The differential expression analysis of CS-Tat vs. EV (empty vector) and CC-Tat vs. EV identified several genes to be significantly enriched (Supplementary Table 2 and Supplementary Figure 4). Of note, the CS and CC-Tat proteins being similar in sequence activated several common signaling pathways, but dysregulated specific pathways differentially. Some of the pathways commonly regulated by both the Tat proteins included metabolic processes of cellular macromolecules, regulation of gene expression, and nucleic acid metabolic processes, among others (Supplementary Table 3). Additionally, CS-Tat enriched for the regulation of the cellular membrane organization and regulation of I-kappaB kinase/NF-kappaB cascade and intracellular receptor-mediated signaling pathways (Supplementary Table 3). Some of the biological pathways significantly enriched by CS-Tat comprised VEGF, ERBB, EGF/EGFR, phosphatidylinositol, and angiopoietin receptor Tie2-mediated signaling. The data of CS-Tat induction collectively implied the involvement of pathways associated with growth factor receptors and angiogenic signaling (Supplementary Table 3). CC-Tat, in contrast, significantly enriched the pathways involved in apoptosis, TLR signaling, cell cycle, Jak-STAT, and p53 signaling (Supplementary Table 3).

### CS-Tat Augments CCL2 Secretion From HUVEC

Since a considerable amount of Tat and Tat-mediated cellular factors are secreted into the conditioned medium (37), we used the conditioned medium of Dox-induced Jurkat-Tat cells to examine the effects of secreted Tat and its secondary mediators on angiogenic responses (38). HUVEC are routinely used in studies investigating angiogenesis (31, 38–41). We used HUVEC cells to compare the angiogenic responses of CS-Tat vs. CC-Tat secreted into the t conditioned media of stable Jurkat cells. The interaction of Tat with the VEGFR-2/KDR or integrins on the cell surface causes the upregulation of chemokines such as CCL2 (monocyte chemoattractant protein, MCP-1) and cytokines such as IL-6 and IL-8 in endothelial cells (34–37).

**Figure 2:**
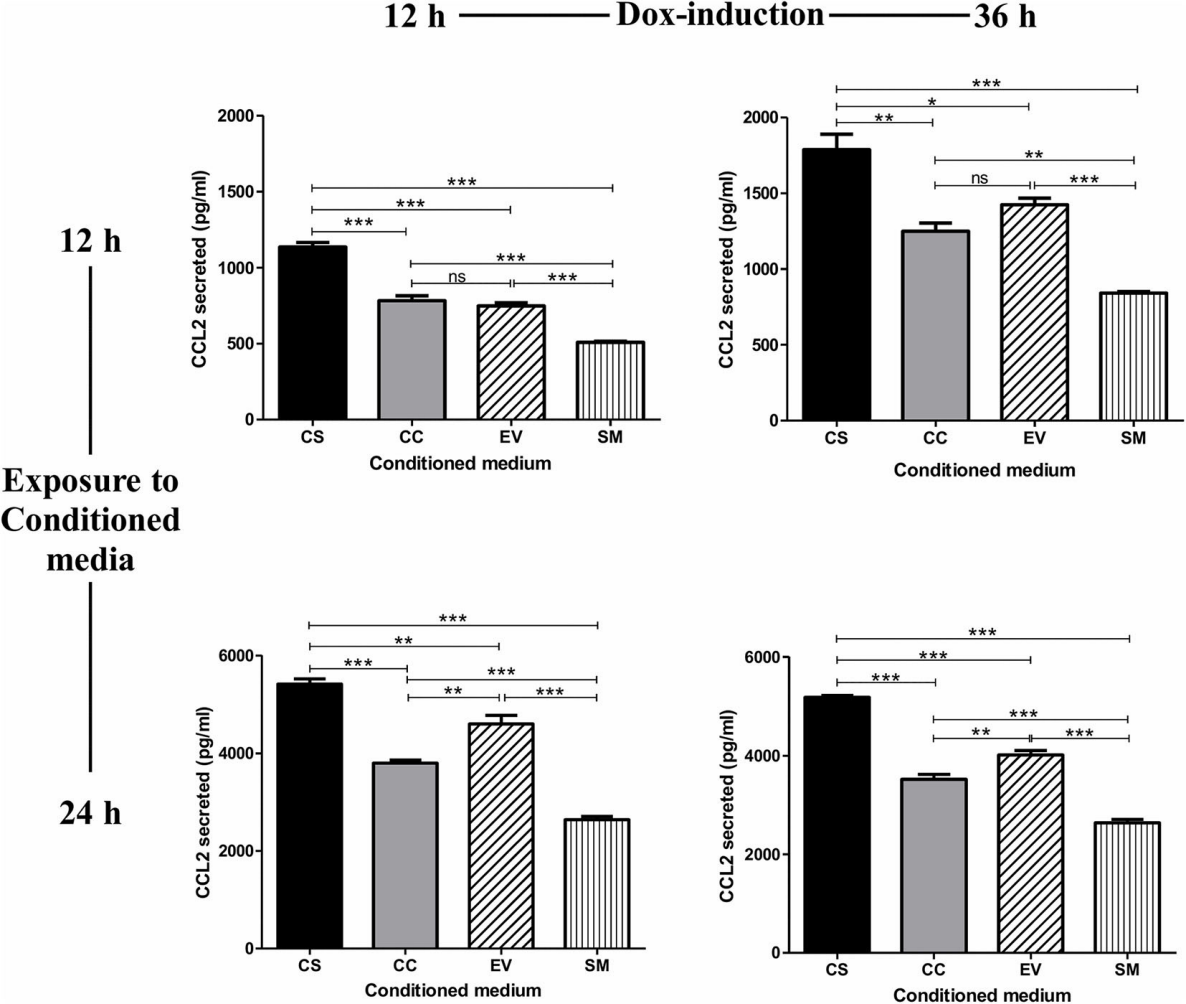
Conditioned medium of CS-Tat Jurkat cells augments CCL2 secretion from HUVEC. The cell-free conditioned media from the Jurkat cells (CS-Tat or CC-Tat or EV) were collected following 12 (left panel) or 36 h (right panel) of Dox induction. The conditioned media were added to HUVEC monolayers, and CCL2 secretion from the HUVEC was determined after 12 (top panel) or 24 h (bottom panel) of exposure to the conditioned media. The SM (HUVEC treated with RPMI supplemented with 10% FBS) served as the control for background CCL2 expression in the assay. The CCL2 levels were evaluated using an antigen-capture ELISA. EV, empty vector control; SM, serum medium control. The data are representative of three independent experiments. One-way ANOVA with Tukey–Kramer post-test (^*^p < 0.05, ^**^p < 0.01, and ^***^p < 0.001).

The treatment of the HUVEC with conditioned medium from CS-Tat Jurkat cells induced a significantly higher level of CCL2 secretion from HUVEC (1137 ± 49.6 pg/ml, Figure 2) as compared to that of CC-Tat (782.5 ± 57.7 pg/ml, *p* < 0.0001) whereas no such differences were found between CC-Tat vs. EV. Since the HUVEC are primary cells, a considerable background level CCL2 secretion, SM (serum media control, 507.8 ± 12.77 pg/ml) was also observed; these levels, however, were significantly low to those of Tat treatment. The profiles of the CCL2 response remained mostly the same and significant regardless of the duration of the dox induction of Jurkat cells or the length of the treatment of the HUVEC cells with the conditioned medium. Since the two Tat proteins, CS and CC-Tat, differ from each other at only one signature amino acid residue (serine 31 vs. cysteine 31) in the cysteine-rich domain (CRD), the differential cytokine induction between the variant forms of Tat could be attributed to this single SAR difference. Of note, we confirmed that the conditioned medium did not contain any CCL2 by using the CS, CC, and EV conditioned media as control samples during the ELISA assay. The absorbance of these control samples was found to be equal to or lower than the assay blank (cell culture media), thus confirming that the CCL2 detected was induced from HUVEC.

**Figure 3:**
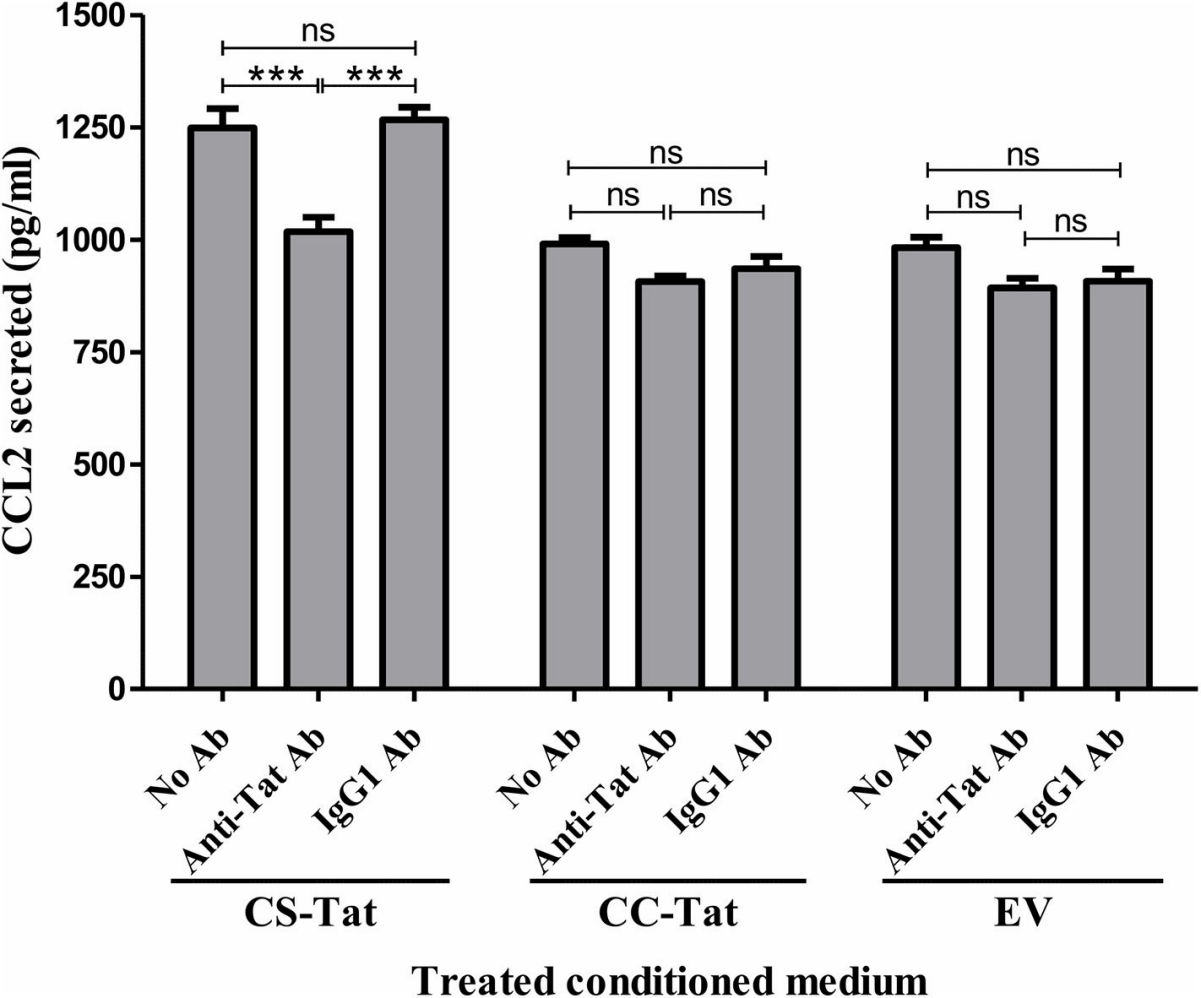
Tat neutralization in the conditioned media attenuates S31-specific differential CCL2 secretion. Conditioned media were collected from Jurkat-Tat cells at 12 h following Dox induction. The conditioned media were incubated with a highly potent anti-Tat monoclonal antibody (1 μg/ml of E2.1 antibody, raised in-house) or an IgG1 isotype control antibody or “no antibody” for 30 min at room temperature with gentle agitation. The HUVEC were exposed to the Tat-neutralized conditioned media for 12 h, and the CCL2 secretion was quantified using ELISA. EV: empty vector control. Two-way ANOVA with Bonferroni post-test (^***^p < 0.001).

To investigate if the differential induction of CCL2 in EC was Tat-specific, the conditioned media were subjected to Tat neutralization and, then, evaluated for CCL2 induction from the HUVEC (Figure 3). The treated conditioned media were added to HUVEC, and the CCL2 secreted into the medium at the end of 12 h was evaluated using an antigen-capture ELISA. Pretreatment of the CS-Tat-conditioned medium with a highly potent anti-Tat monoclonal antibody, but not an isotype control, reduced the secretion of CCL2 significantly (Figure 3). The mean CCL2 levels in the medium dropped from 1248 ± 74.64 pg/ml to 1018 ± 56.25 pg/ml following Tat neutralization, a level comparable to that of the CC-Tat medium (990.3 ± 24.33 pg/ml). In summary, although the influence of the other host factors in the conditional medium cannot be ruled out, the data of Tat neutralization confirms a significant role played by CS-Tat in inducing a significantly elevated concentration of CCL2.

The levels of CCL2 induced by the EV conditioned medium were significantly higher as compared to SM under most test conditions. Jurkat cells exhibit a basal level of an activated phenotype in culture, which can further be enhanced in response to several stimuli. The rtTA3Tet-On system engineered into EV Jurkat cells could also contribute to the background noise if activated by the cellular transcriptional noise. Of note, in our experiments, we observed comparable levels of CCL2 secretion by the conditioned media from parental EV cells as well as that of CC-Tat Jurkat cells, suggesting background noise.

### The CS-Tat Conditioned Media Exert a Superior Tube-Formation Response in HUVEC

**Figure 4:**
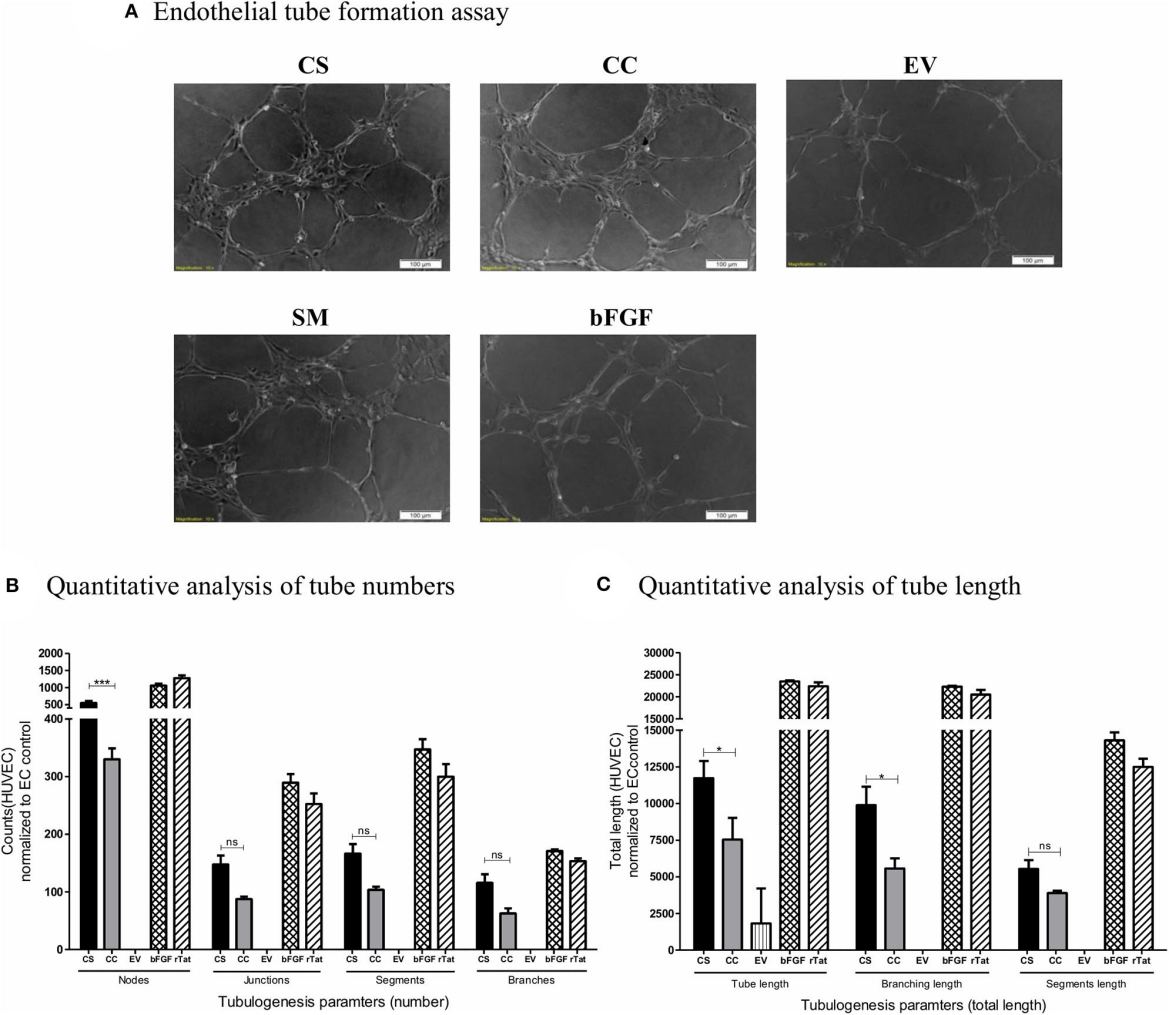
Tube formation of HUVEC following treatment with conditioned media. Conditioned media from Jurkat-Tat cells were harvested following 12 h of Dox-induction. 20,000 HUVEC in respective conditioned media were seeded over Matrigel in a 96-well plate. The HUVECs seeded in RPMI supplemented with 10% FBS served as the endothelial cell (SM) background control, and HUVECs treated with bFGF (2 ng/ml) and rTat (1 ng/ml) served as positive controls in the assay. **(A)** The cells were imaged at 2 h intervals for the tube formation, and the images captured at 4 h using a 10× objective are presented. The images were quantified using the Angiogenesis Analyzer plugin for ImageJ (NIH). The images were transformed to skeletonized binary tree form and analyzed for the tube parameters. The individual values were normalized to the SM control values for each parameter. **(B)** The normalized counts for the number of nodes, junctions, segments, and branches for CS, CC, EV, and bFGF treated HUVECs. **(C)** The total length, branching length, and segment length following different treatments. The counts and lengths ± SD are plotted, and the data are representative of three independent experiments. Two-way ANOVA with Bonferroni post-test (^*^p < 0.05, ^***^p < 0.001 and ns, non-significant).

We exposed HUVEC seeded on the Matrigel in a 96-well plate in triplicate wells to different conditioned media (CS-Tat, CC-Tat, and EV Jurkat conditioned media, 12 h dox induction) and measured the morphological features. Among the parameters evaluated, the number of junctions, segments, and branches did not show any significant difference between the treatment with the conditioned media of CS-Tat and CC-Tat. Importantly, however, we observed a significantly higher number of nodes in the CS-Tat treatment as compared to that of CC-Tat (545.33 ± 98.04 and 330 ± 32.7, respectively, *p* < 0.001; Figure 4A, and Supplementary Figure 5). We also found significantly longer tubes induced by CS-Tat as compared to CC-Tat (11,734.67 ± 2,017.9 and 7,546.66 ± 2,553.6, respectively, *p* < 0.05; Figure 4B). Additionally, the branching length of tubules for CS-Tat was significantly higher than that of CC-Tat (p < 0.05, Figure 4C). The response of HUVEC to rTat (purified recombinant Tat of HIV-1C origin) in different tubulogenesis parameters was comparable to that of the bFGF-positive control asserting the Tat-specific nature of the induction. Of note, the length of tubule segments induced by CS-Tat and CC-Tat did not differ significantly. Since tube formation assay serves as a robust measure of angiogenic potential, the above observations, when combined with the earlier finding of higher levels of expression of CCL2, allude to a superior angiogenic potential of CS-Tat that could be ascribed to the presence of the SAR S_31_ in C-Tat.

### CS-Tat Conditioned Medium Stimulates Enhanced Cell Migration, Trans-Endothelial Permeability, and Monocyte Adhesion

CCL2, being a potent chemokine, can recruit monocytes and T-cells and augment their adhesion and transmigration across the endothelial monolayer (42, 43). We, therefore, explored if the augmented CCL2 secretion by CS-Tat can induce enhanced activation, migration, and invasion of endothelial cells and promote the migration of monocytes and T-cells across the endothelial monolayer.

**Figure 5:**
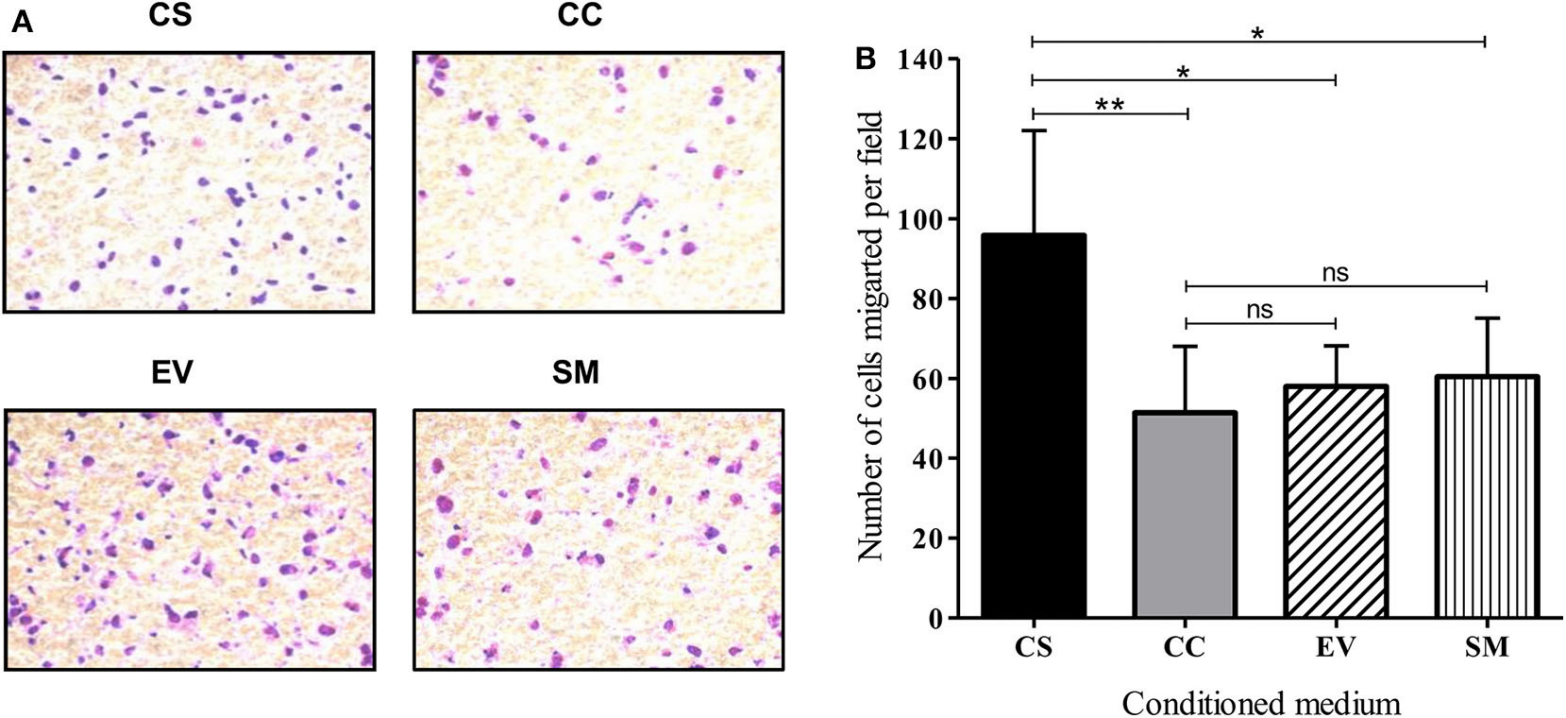
CS-Tat stimulates superior migration and invasion of HUVEC following an exposure to Jurkat-Tat-conditioned media. **(A)** HUVECs suspended in serum-free media were seeded in Matrigel (300 μg/ml) coated 8 μm cell culture inserts at a density of 0.5 × 10^6^ cell/ml. The receiver wells contained conditioned media as labeled. HUVEC seeded in RPMI supplemented with 10% FBS served as the background (SM) control. The cells that did not migrate were scraped off using cotton swabs. The cells that migrated toward the lower chamber after 12 h of incubation were fixed, permeabilized, and stained with Giemsa for imaging using a 20× objective. **(B)** Five fields per treatment were imaged and the number of migrated cells were counted using ImageJ. The mean numbers of cells per field ± SD were plotted for each treatment. Conditioned media from Jurkat cells were harvested at 12 h post-dox induction. The data are representative of three independent experiments. One-way ANOVA with Tukey–Kramer post-test (^*^p < 0.05, ^**^p < 0.01 and ns, non-significant).

To evaluate the migration and invasive behavior of HUVEC, the cells were suspended in serum-free medium and seeded on Matrigel-coated transwell inserts. The mean number of cells migrated per field under the influence of CS-Tat conditioned medium (95.8 ± 26.2) was significantly higher as compared to that of CC-Tat (51.4 ± 16.6, *p* < 0.01) or as compared to the EV and SM controls (Figures 5A,B, and Supplementary Figure 6). Since the migration of cells across the porous membrane necessarily requires the cells to migrate and invade through the matrigel, the assay takes into account both the invasive and migratory potential of cells.

**Figure 6:**
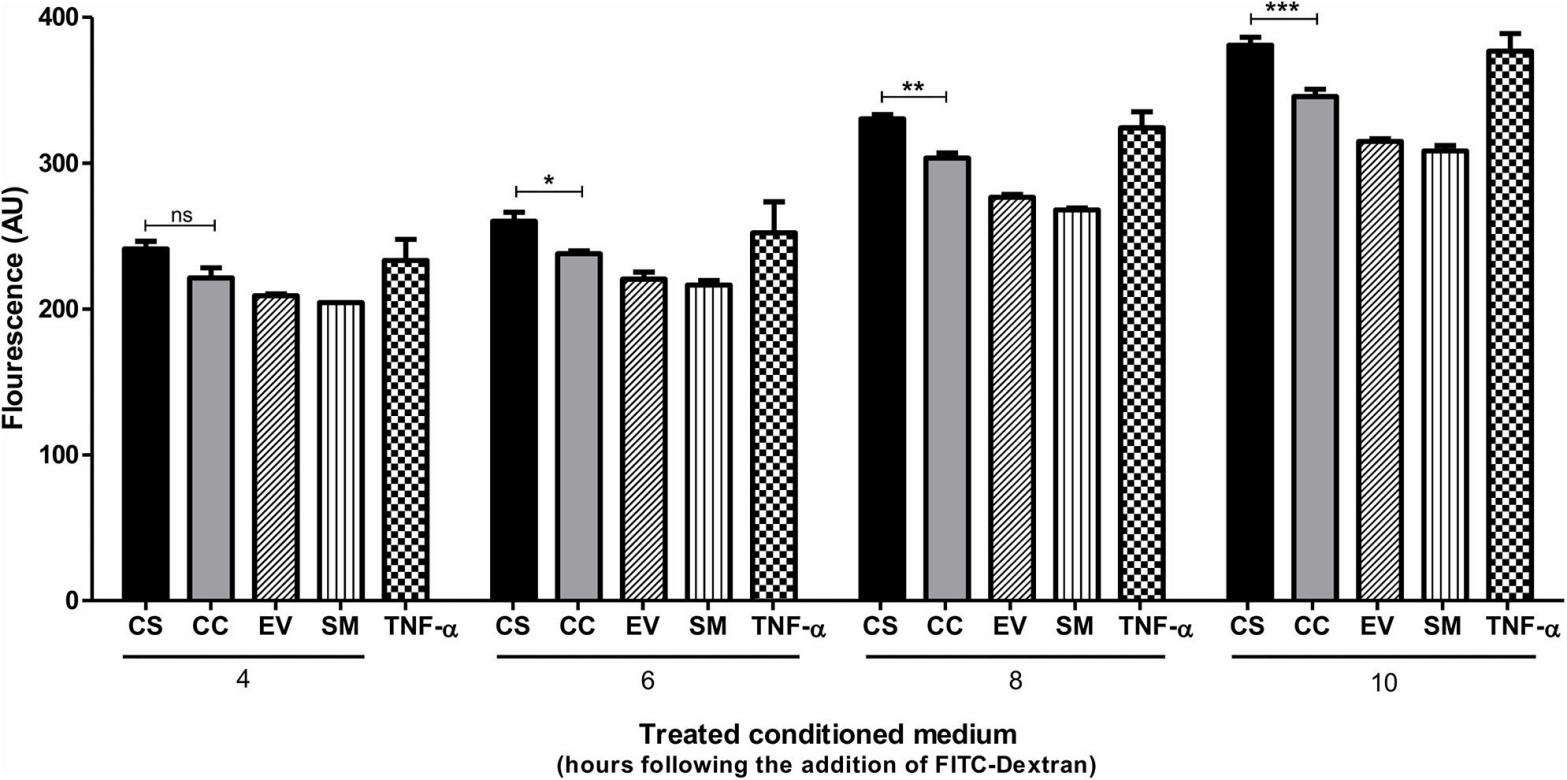
CS-Tat enhances trans-endothelial permeability of FITC-Dextran across the endothelial monolayer. hCMEC/D3 cells were seeded on collagen-coated 0.4-μm culture inserts and allowed to grow to confluence. The cells were serum-starved for 12 h before the assay. Jurkat-Tat-conditioned media collected at 12 h following dox induction were added to the confluent hCMEC/D3 monolayer on the inserts and the wells. Following 24 h of incubation at 37°C, a fresh medium supplemented with FITC dextran (0.1 μg/ml) was added apically to the inserts. Culture media were collected from the wells at regular intervals and monitored for fluorescence. hCMEC/D3 cells treated with 10 ng/ml of TNF-α served as the positive control in the assay. The data are representative of three independent experiments. Two-way ANOVA with Bonferroni post-test was used for the statistical evaluation (^*^p < 0.05, ^**^p < 0.01, ^***^p < 0.001 and ns, non-significant).

In addition to serving as an inflammatory mediator, CCL2 serves as a potent chemokine modulating the expression levels of several cell surface adhesion molecules and junctional adhesion molecules, thus altering the trans-endothelial permeability properties of the blood–brain barrier (BBB) (44–46). To this end, we examined if the enhanced CCL2 response of CS-Tat treatment can translate into augmented trans-endothelial permeability. A confluent monolayer of hCMEC/D3 cells, extensively used as an *in vitro* model for BBB, seeded on collagen-coated 0.4-μm culture inserts served as a model for the BBB. We monitored the fluorescence intensity of FITC-dextran in culture media at regular intervals. CS-Tat conditioned media caused an increased FITC-dextran 40 flux across the monolayer (260.3 ± 6 and 237.9 ± 1.9 AU, respectively, for CS- and CC-Tat at the 6 h time point, *p* < 0.01; Figure 6). Of note, the data are consistent at different durations of treatment with the conditioned media. The disruption of the BBB was pronounced and highly significant as the duration of the treatment increased (380.9 ± 5.4 and 345.7 ± 5.1 AU, respectively, for CS and CC-Tat at the 10 h time point, *p* < 0.001).

**Figure 7:**
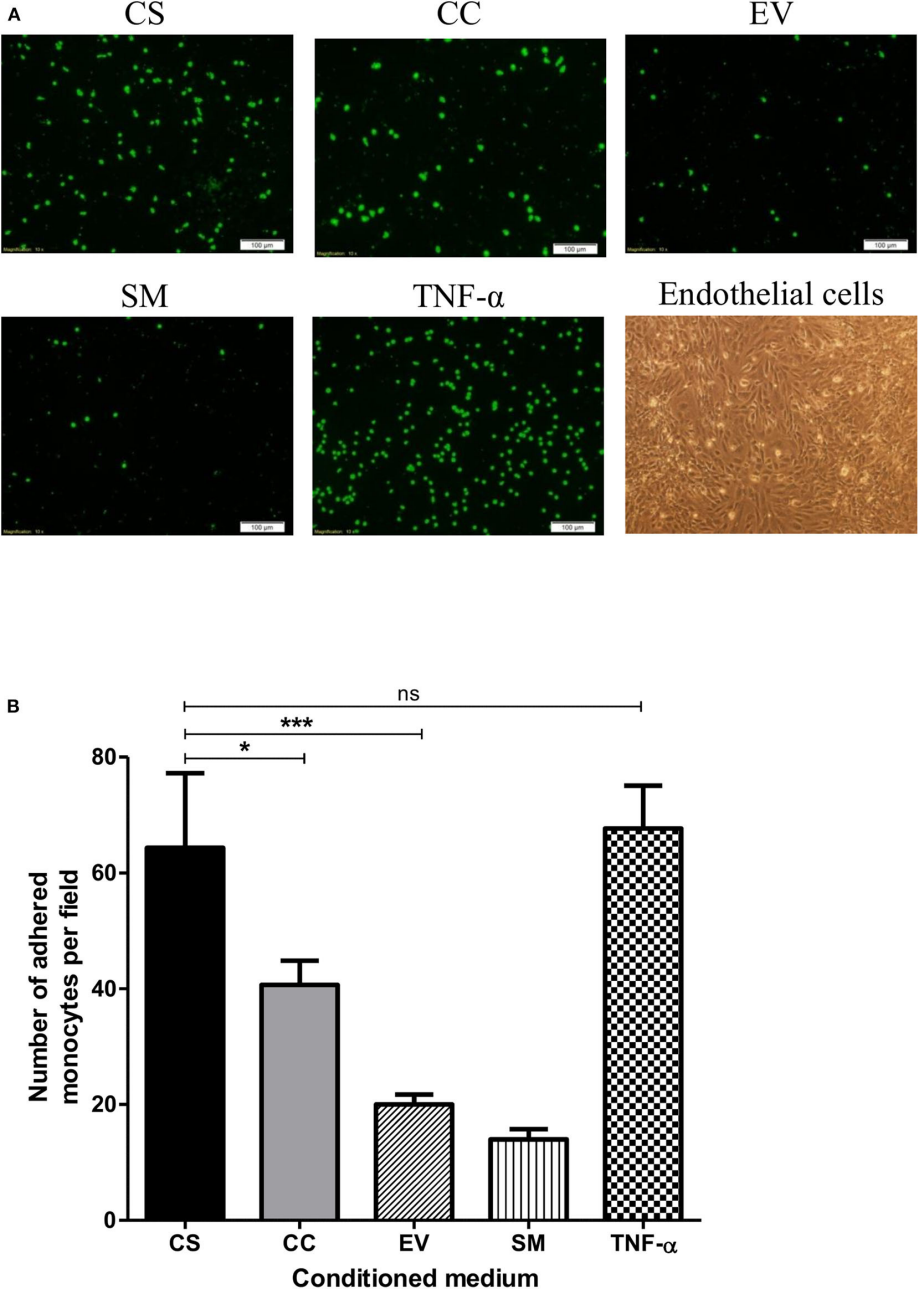
The treatment with CS-Tat Jurkat-conditioned medium augments monocyte-endothelial cell adhesion. hCMEC/D3 endothelial cells were grown to confluence on collagen-coated coverslips in 24-well plates. Following 12 h of serum starvation, the monolayer was exposed to Jurkat-Tat-conditioned media for 24 h. In parallel, THP1 cells were treated with the Jurkat-Tat conditioned media for 12 h, washed, and labeled with CalceinAM. The CalceinAM-labeled THP1 cell suspension was added to the endothelial monolayer, and following 10 h of the addition of THP1 cells, the coverslips containing the endothelial monolayer were washed with HBSS. **(A)** The adhered THP1 cells under each treatment were imaged using a fluorescent microscope. Endothelial cells treated with RPMI supplemented with 10% FBS served as the background control (SM) and cells treated with 10 ng/ml of TNF-α served as the positive control in the assay. **(B)** Five fields per treatment were used for the quantification, and the number of adhered monocytes per field were counted using ImageJ. The mean numbers of cells per field ± SD were plotted for each treatment. The assay was repeated twice, one-way ANOVA with Tukey–Kramer post-test (^*^p < 0.05, ^***^p < 0.001 and ns, non-significant).

HIV-1 Tat augments the expression of the integrin adhesion molecules (β2 integrins) on monocytes and T-cells and enhances their adhesion to endothelial cells (47, 48). To this end, we asked if exposing the hCMEC/D3 and THP1 cells to different Tat-conditioned media would lead to differential adhesion of monocytes to hCMEC/D3 cells. The adhesion of CalceinAM-labeled THP1 cells was monitored using a fluorescent microscope (Figure 7A). CS-Tat treatment resulted in a significantly higher level of cell adhesion as compared to that of CC-Tat, or the SM or the EV controls (64.33 ± 12.9, 40.67 ± 4.2, 20 ± 1.7, 14 ± 1.7, respectively, *p* < 0.05; Figure 7B).

## Discussion

Several groups, including ours, have previously demonstrated the crucial functions SAR may play in governing the biological properties of Tat (11, 37, 49, 50). Tat-mediated modulation of gene expression in cells of diverse lineage has been investigated extensively (10, 51–53). Using primary cultures and neuronal cell lines, Chang et al. examined the impact of HIV-1 Tat on the expression of miRNAs and demonstrated that Tat deregulates the levels of several miRNAs, including miR-34a, the most highly induced miRNAs in Tat-treated neurons (10). Evaluating gene expression modulation by Tat in H9-Tat cells using cDNA microarray technology, de la Fuente et al. reported Tat-mediated down-modulation of cell surface receptors, co-receptors including those associated with cellular receptor tyrosine kinase (RTK) activity and transcription coactivators such as p300/CBP and SRC-1 (51). Gibellini et al. performed a cDNA-membrane-array using CD4^+ve^ Jurkat cells constitutively expressing Tat under serum-starved conditions and demonstrated 2- to 3-fold upregulation of several cellular genes including transcription factors, cellular receptors, adaptors, and mediators of signal transduction pathways (52). Given the highly diverse conditions of experimentation, including stable vs. transient expression of Tat, the lineage of the host cell, and the endpoint evaluation, the conclusions drawn are extremely variable making it difficult to draw unifying themes on Tat-mediated modulation of host cell transcription.

The present study is unique in three different qualities. First, unlike the previous studies, all of which employed Tat of HIV-1B origin, the present study used HIV-1C Tat. Given the subtype-specific molecular differences and SAR, an examination of viral factors of diverse genetic backgrounds is likely to throw more light on the biological differences among viral families. Second, in the present study, we used two different Tat proteins that are discordant for a single SAR at position 31—comparing serine vs. cysteine at this location. Our research, thus, is designed not only to examine the impact of Tat expression on the landscape of the host factors but also to compare the effect of Tat proteins discordant for a single amino acid residue. Lastly, unlike the previous studies, our work exercised control on Tat expression crucial to examining the earliest and direct targets of Tat, using RNA-Seq. The use of RNA-Seq gave the advantage of unbiased and easy identification of rare and low-abundant transcripts with increased sensitivity and specificity. One of the technical limitations of the present work is the absence of a primary cell model. Given that the study design required the establishment of double stable cells for controlled Tat expression, extending this work to primary CD4+ T-cells would be technically challenging. Of note, the role of SAR S31 in C-Tat has been examined in the absence of other viral proteins and regulatory elements. Whether the findings of the present analysis would be consistent in the context of full-length viral strains needs to be determined.

The present study stemmed from our earlier publication, which identified S_31_ to be a determinant of the defective chemokine function of HIV-1C Tat (11). It is intriguing, however, to find that C-Tat despite being a defective chemokine induces an enhanced CCL2 chemokine response from the endothelial cells. The exposure of endothelial cells to Tat causes a time- and dose-dependent release of CCL2 into the culture supernatant (42, 54). In the light of the salient functions of CCL2 as a chemokine, combined with its association with endothelial dysfunction and disruption of the BBB, it would be of interest to examine if the enhanced CCL2 response from EC following CS-Tat treatment would also translate into an altered expression of lymphocyte, monocyte, or endothelial cell surface adhesion molecules and increased expression of Matrix metalloproteinases (MMPs). Recent research has suggested an increase in virus production in the presence of CCL2 in HIV-infected macrophages, and this effect is seen to be Gag-mediated via the LYPX motif. This observation is seen to be subtype-specific, where in sharp contrast to HIV-1B, HIV-1C fails to show this response due to the absence of the LYPX motif (55). Thus, the CS-Tat-induced CCL2 production may influence biological functions other than the viral replication of HIV-1C.

The demonstration that S_31_ in CS-Tat predisposes Tat toward enhanced endothelial cell activation is a novel finding of the present study. The RNA-Seq analysis of CS-Tat revealed the activation of several signaling pathways, broadly suggesting a significantly modulated angiogenesis-related signaling process. In addition to the enhanced CCL2 induction in HUVEC, the CS-Tat-conditioned medium caused enhanced cell migration and invasion of endothelial cells. These observations, along with the *in vitro* tube formation results, provided convincing evidence to an angiogenic signaling program induced by the CS-Tat Jurkat-conditioned medium in HUVEC. The pro-angiogenic properties of extracellular HIV-1B Tat, especially in association with several proinflammatory cytokines, have been elegantly reviewed (56). The integrity of the arginine–glycine–aspartic acid (RGD) motif and the basic domain is critical for Tat to exert growth-promoting properties on the endothelial cells by binding to the α5β1 and αvβ3 integrins (38, 56–58). A vast majority of HIV-1C viral strains do not contain the RGD motif in exon 2 due to a natural variation. It would be necessary to understand if the natural variation of C31S in HIV-1C Tat, leading to significantly enhanced induction of CCL2 production, is a compensatory mechanism for the differences in the basic domain and the RGD motif.

Furthermore, HIV-associated cardiovascular diseases account for approximately 10% of the deaths in HIV-positive patients (59). Increased adhesion of leukocytes to the aortic epithelium and endothelial dysfunction is a pathogenic manifestation commonly observed in HIV-associated microvascular and angio-proliferative disorders (60). Since EC have not been reported to support HIV-1 replication, the observed vascular dysfunction is believed to be mediated by molecules released from HIV-infected cells, including viral proteins and induced cellular factors (61). Tat-mediated activation of EC associated with the enhanced monocyte adhesion to EC is mediated via the activation of MAP kinases and NF-κB (62). Additionally, Tat stimulates the upregulation of adhesion molecule ICAM-1 by downregulating miR-221 in an endothelial-specific manner. The HIV-1 Tat protein alongside inflammatory cytokines, synergistically enhances endothelial cell growth, resulting in the induction of angiogenic Kaposi's sarcoma like lesions (57, 58, 63, 64). Of note, the prevalence of Kaposi's sarcoma is rare in HIV-1C infection (65). The available data implicates a significant role for Tat in HIV-associated cardiomyopathies (62, 66). In the context of the present study, the evaluation of cardiomyopathy and KS cases in the HIV-1C-infected population becomes essential. Additionally, further research is required to evaluate the mechanistic details of the cellular pathways involved in the CS-Tat-mediated angiogenic response in EC.

In summary, the findings from the present study are likely to help appreciate the functional significance of the SAR residues influencing the unique biological properties of diverse genetic families of HIV-1. The ability of CS-Tat to induce an activated phenotype in endothelial cells conferring on them enhanced EC migration, invasion, and *in vitro* morphogenesis is significant, especially in the context of HIV-associated neuronal and cardiovascular disorders.

## Data Availability Statement

The datasets generated for this study can be found in the NCBI SRA accession number SUB7165990, http://www.ncbi.nlm.nih.gov/geo/query/acc.cgi?acc=GSE89266.

## Author Contributions

MM: conceptualization, data curation, investigation, validation, writing original draft, and reviewing and editing of the article. KB, MV, RB, and RS: resource provision, data curation, and validation. UR: conceptualization, fund acquisition, validation, writing, reviewing, and editing of the article. All authors contributed to the article and approved the submitted version.

## Conflict of Interest

RB, RS, KB, and MV were employed by company Bionivid Pvt. Ltd. The remaining authors declare that the research was conducted in the absence of any commercial or financial relationships that could be construed as a potential conflict of interest.
