# Supplementary material for: The Signature Amino Acid Residue Serine 31 of HIV-1C Tat Potentiates an Activated Phenotype in Endothelial Cells
Source: Front Immunol. 2020 Sep 25;11:529614. doi: 10.3389/fimmu.2020.529614 (PMC7546421; doi:10.3389/fimmu.2020.529614)
Supplement: Supplementary file 4 [file Data_Sheet_1.PDF]

(A) Fluorescence-assisted cell sorting of transduced rtTA3-Jurkat

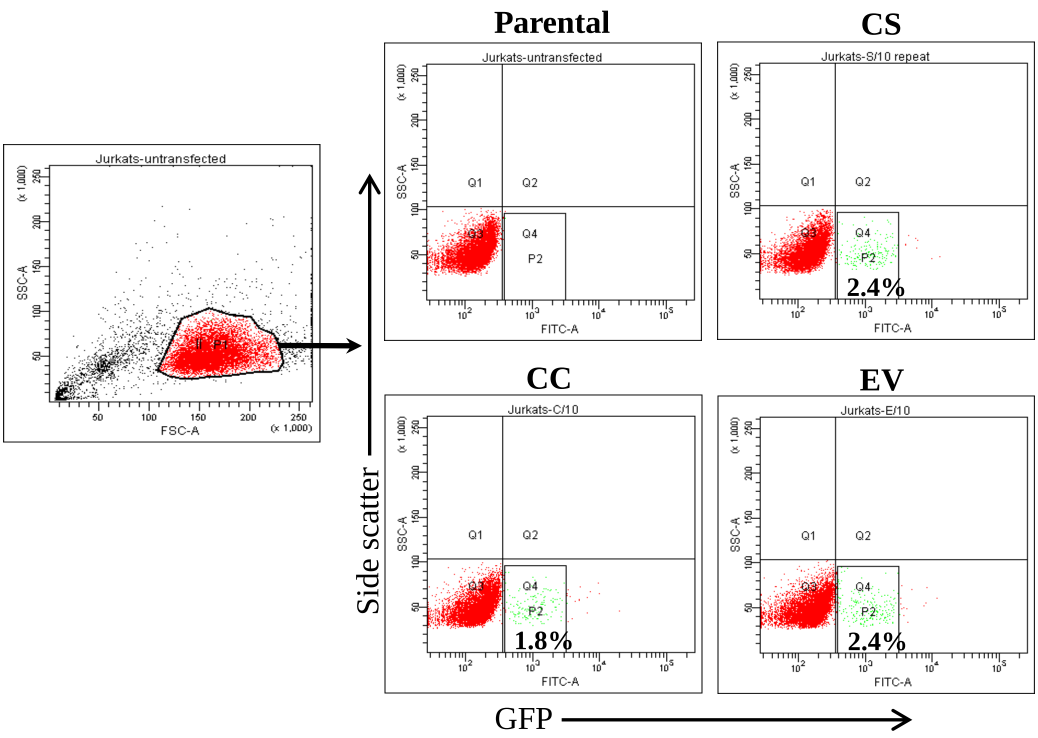

(B) Frequency of integration

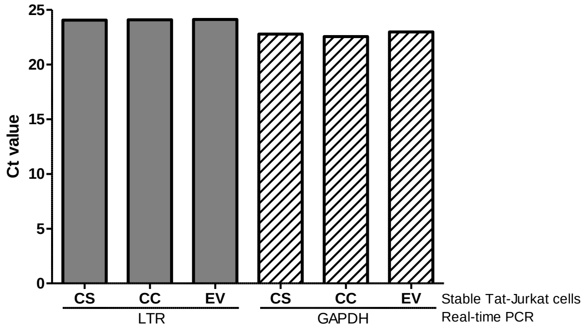

Supplementary Figure 1: Establishing CS, CC and EV Jurkat stable cells using Fluorescence-assisted cell sorting. **(A)** The transduced Jurkat cells were allowed to stabilize for ten days following infection. The cells were subsequently analyzed using a Flow cytometer for copGFP expression. The flow cytometry plots for the parental (untransduced) Jurkat cells and CS-Tat, CC-Tat, EV pseudotyped virus-transduced Jurkat cells are presented. The cell populations expressing GFP (gated population P2 in Q4) were sorted for each virus and used in the subsequent analyses. **(B)** A comparative analysis of the events of integration in the stable Tat-Jurkat cells: Ten ng of genomic DNA isolated from the cells were amplified in a real-time PCR using primers targeting the strong-stop negative-strand DNA in the viral LTR. GAPDH amplification was used for normalization. The data are representative of two independent experiments. The comparable Ct values are suggestive of equivalent frequency of integration in the different Tat-Jurkat cells.
