# Supplementary material for: The Signature Amino Acid Residue Serine 31 of HIV-1C Tat Potentiates an Activated Phenotype in Endothelial Cells
Source: Front Immunol. 2020 Sep 25;11:529614. doi: 10.3389/fimmu.2020.529614 (PMC7546421; doi:10.3389/fimmu.2020.529614)
Supplement: Supplementary file 5 [file Data_Sheet_2.PDF]

(A) Time-dependent modulation of gene expression in CC-Tat Jurkat cells

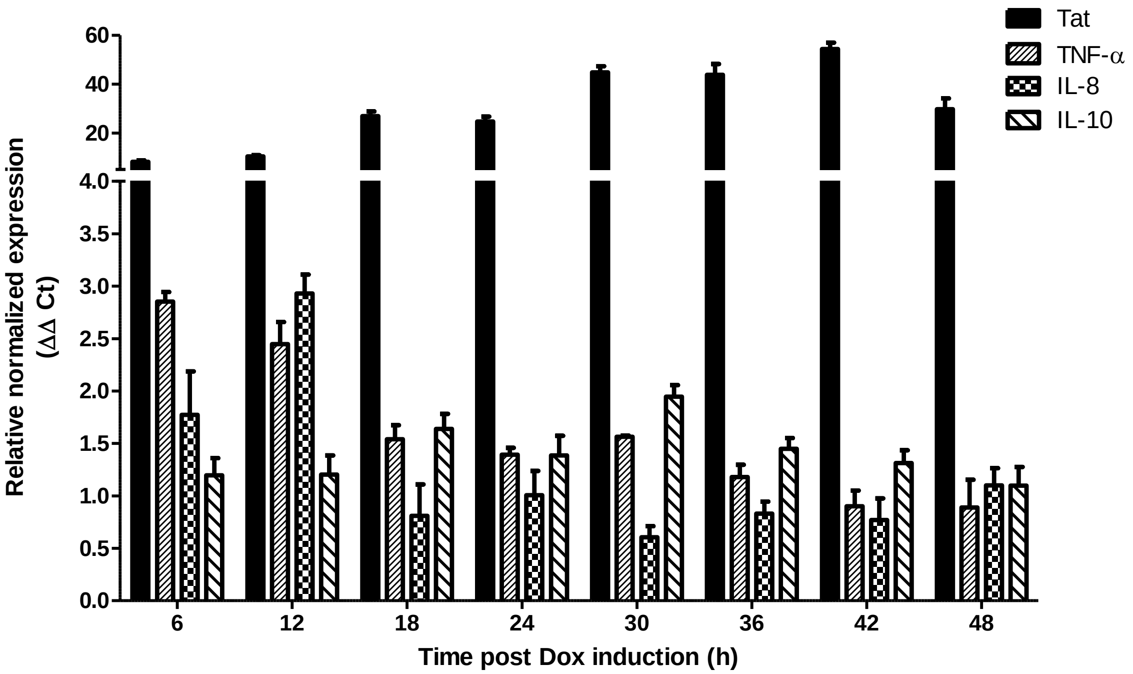

(B) Tat transcript expression

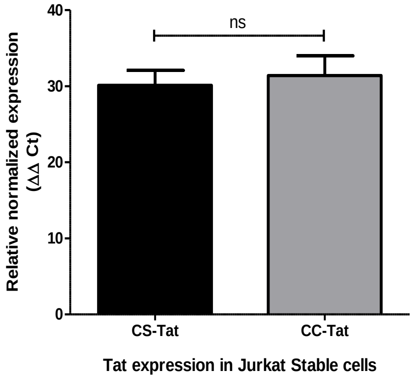

(C) Cytokine transcript expression

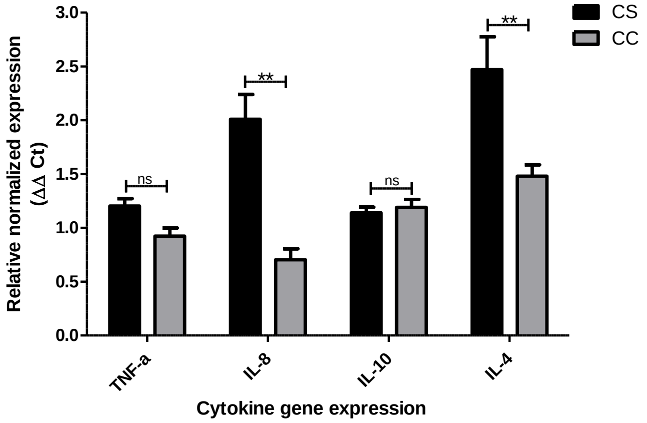

Supplementary Figure 2: Quantitative real-time PCR analysis of Tat gene expression and a few cytokine genes following Dox-induction. (A) The rtTA3-CC-Tat Jurkat cells were seeded at a density of 0.6x106 cells/ml and treated with 800 ng/ml of Doxycycline. The cells were harvested at 6 h intervals, total RNA was isolated, 2 μg of RNA was converted to cDNA, and five μl of 10-fold diluted cDNA was used as template for the Real-time PCR, using primers specific for Tat or a few cytokine genes (TNF-α, IL-8 or IL-10). GAPDH was used as a reference gene control for normalization and the relative normalized gene expression values (determined using the ΔΔCt method) were plotted against time for each gene. The 12 h time point was selected for the subsequent analyses. **(B)** Tat and **(C)** Cytokine gene-expression profiles of CS-Tat Vs. CC-Tat, 12 h following Dox-induction. The relative normalized mean values ± SD are plotted. The data are representative of three independent experiments. Unpaired two-tailed t-test and one-way ANOVA with Tukey-Kramer post-test was used for the statistical evaluation (**p<0.001 and ns, non-significant).
