# Supplementary material for: The Signature Amino Acid Residue Serine 31 of HIV-1C Tat Potentiates an Activated Phenotype in Endothelial Cells
Source: Front Immunol. 2020 Sep 25;11:529614. doi: 10.3389/fimmu.2020.529614 (PMC7546421; doi:10.3389/fimmu.2020.529614)
Supplement: Supplementary file 6 [file Data_Sheet_3.PDF]

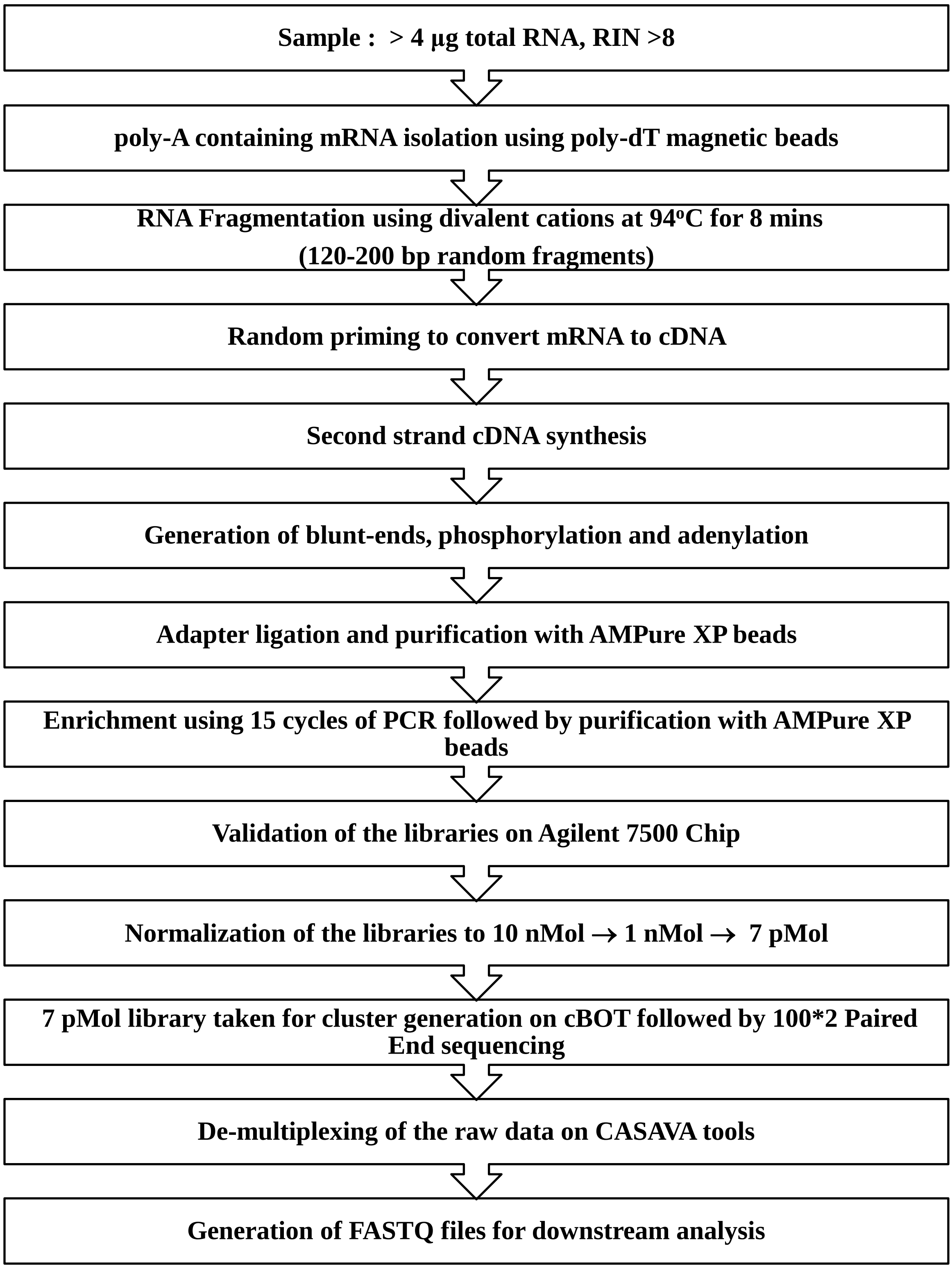

Supplementary Figure 3: A flow chart representing the major steps of protocol in the RNA-Seq library preparation .
