# Supplementary material for: The Signature Amino Acid Residue Serine 31 of HIV-1C Tat Potentiates an Activated Phenotype in Endothelial Cells
Source: Front Immunol. 2020 Sep 25;11:529614. doi: 10.3389/fimmu.2020.529614 (PMC7546421; doi:10.3389/fimmu.2020.529614)
Supplement: Supplementary file 7 [file Data_Sheet_4.PDF]

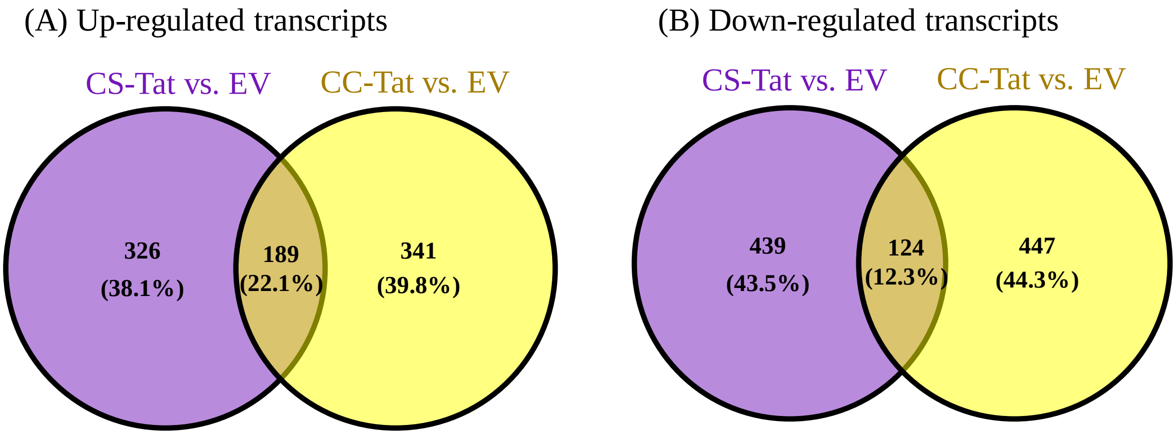

Supplementary Figure 4: Venn Diagram of common and unique dysregulated transcripts in the RNA-Seq data. The Venn diagrams show the differential expression profile under the categories of common and specific (A) up-regulated and (B) down-regulated transcripts in CS vs. CC-Tat differential expression analysis. The number of transcripts and their percentage representation (in parenthesis) under each category are depicted within the Venn diagram.
