# Supplementary material for: The Signature Amino Acid Residue Serine 31 of HIV-1C Tat Potentiates an Activated Phenotype in Endothelial Cells
Source: Front Immunol. 2020 Sep 25;11:529614. doi: 10.3389/fimmu.2020.529614 (PMC7546421; doi:10.3389/fimmu.2020.529614)
Supplement: Supplementary file 8 [file Data_Sheet_5.PDF]

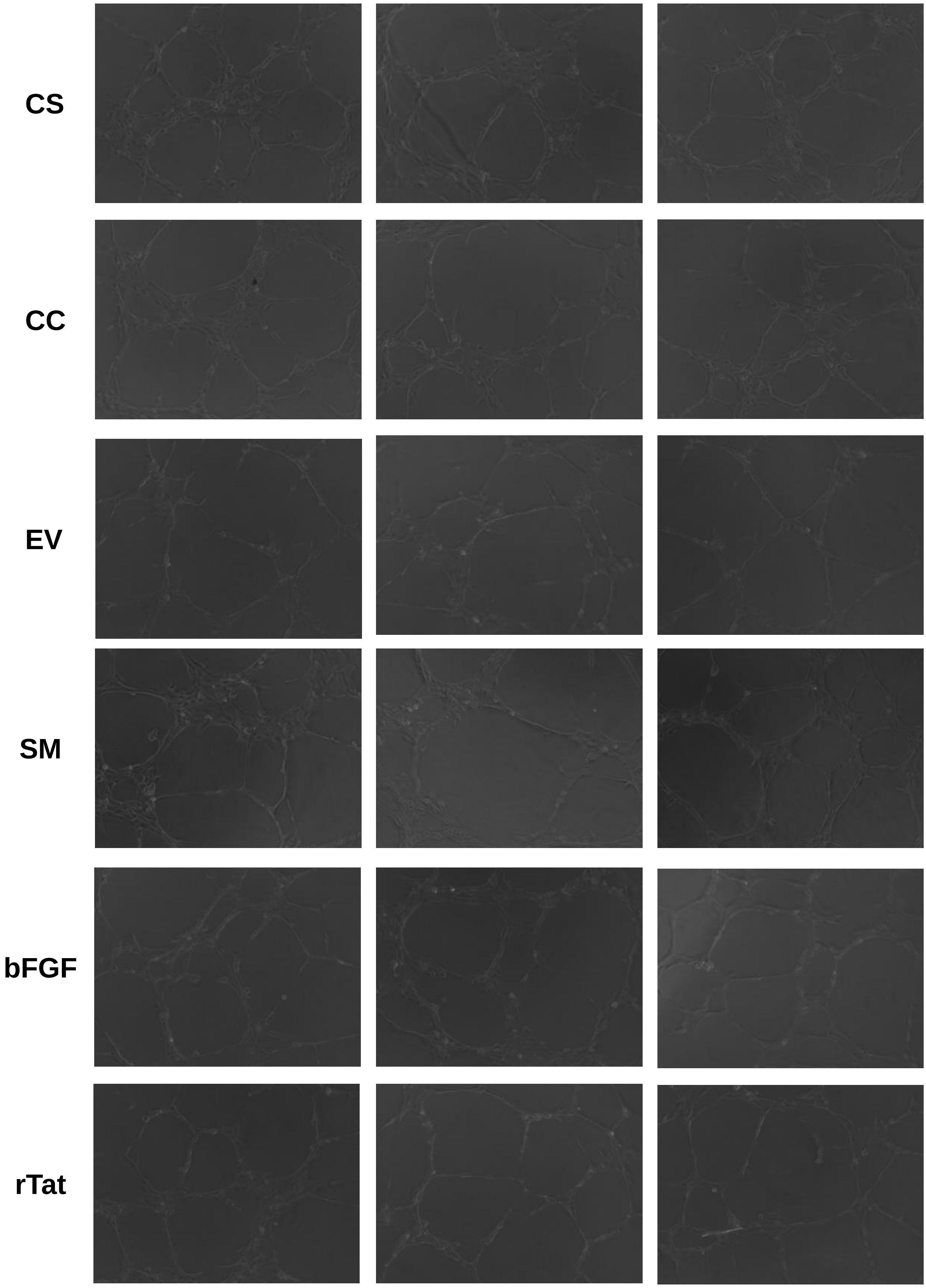


**Supplementary Figure 5: Replicate images used for quantitation of tube formation assay in HUVEC following treatment with conditioned media**. The cells were imaged at 2 h intervals for the tube formation, and the images captured at 4 h using a 10x objective are presented. The images were quantified using the Angiogenesis Analyzer plugin for ImageJ (NIH).
