# Supplementary material for: The Signature Amino Acid Residue Serine 31 of HIV-1C Tat Potentiates an Activated Phenotype in Endothelial Cells
Source: Front Immunol. 2020 Sep 25;11:529614. doi: 10.3389/fimmu.2020.529614 (PMC7546421; doi:10.3389/fimmu.2020.529614)
Supplement: Supplementary file 9 [file Data_Sheet_6.PDF]

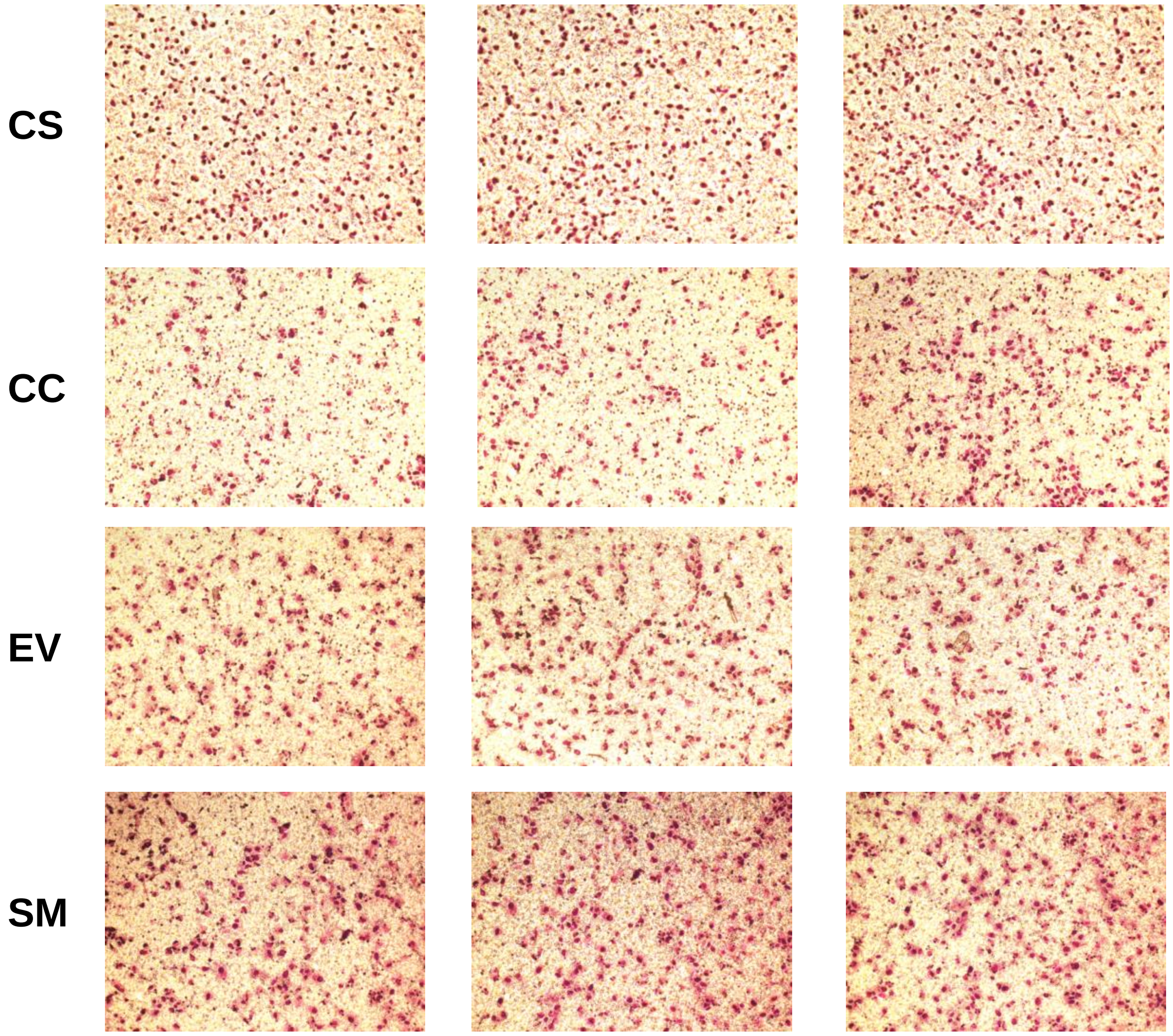

Supplementary Figure 6: Replicate images used for the quantitation of migration and invasion assay in HUVEC following an exposure to Jurkat-Tat conditioned media. HUVECs suspended in serum-free media were seeded in Matrigel (300 µg/ml) coated 8 µm cell culture inserts at a density of 0.5x106 cell/ml. The receiver wells contained conditioned media as labelled. HUVEC seeded in RPMI supplemented with 10% FBS served as the background (SM) control. The cells that did not migrate were scraped off using cotton swabs. The cells that migrated towards the lower chamber after 12 h of incubation were fixed, permeabilized, and stained with Giemsa for imaging using a 10x objective.
